# Prescreening bacterial colonies for bioactive molecules with *Janus* plates, a SBS standard double-faced microbial culturing system

**DOI:** 10.1007/s10482-012-9746-7

**Published:** 2012-05-05

**Authors:** Marina Sánchez-Hidalgo, Javier Pascual, Mercedes de la Cruz, Jesús Martín, Gary S. Kath, Janet M. Sigmund, Prakash Masurekar, Francisca Vicente, Olga Genilloud, Gerald F. Bills

**Affiliations:** 1 Fundación MEDINA, Centro de Excelencia en Investigación de Medicamentos Innovadores en Andalucía, Avda. del Conocimiento 3, Parque Tecnológico de Ciencias de la Salud, 18100 Armilla, Granada, Spain; 2 Design-To-Prototype LLC, 2671 Sky Top Drive, Scotch Plains, NJ 07076 USA; 3 Natural Products Discovery Institute, 3805 Old Easton Road, Doylestown, PA 18902 USA; 4 Department of Plant Biology & Pathology, Rutgers University, Foran Hall, Cook Campus 59 Dudley Rd., New Brunswick, NJ 08901 USA

**Keywords:** Agar-diffusion assay, Agar-overlay assay, Antibiotic, Automation, Katanosins, Lutri plate, *Lysobacter*, Perturbagens, Secondary metabolites

## Abstract

Despite the availability of many culture-based antibiotic screening methods, the lack of sensitive automated methods to identify functional molecules directly from microbial cells still limits the search for new biologically active compounds. The effectiveness of antibiotic detection is influenced by the solubility of the assayed compounds, indicator strain sensitivity, culture media and assay configuration. We describe a qualitative high throughput screening system for detecting cell-perturbing molecules from bacterial colonies employing two opposed agar layers sequentially formed in prototype Society for Biomolecular Screening (SBS) plates, named *Janus* plates. Direct assay of microbial colonies against target organisms in opposed agar layers overcomes some of the limitations of agar overlay methods. The system enables the rapid detection of extracellular cell-perturbing molecules, e.g., antibiotics, excreted directly from environmental isolates. The source bacterial colonies remain separate from the target organism. The growth layer is prepared and grown independently, so environmental strains can be grown for longer intervals, at temperatures and in media that favor their growth and metabolite expression, while the assay layer with pathogens, usually requiring nutrient-rich medium and elevated temperatures, are added later. Colonies to be tested can be precisely arrayed on the first agar surface, thus avoiding dispersion and disturbance of potential antibiotic-producing colonies by overlaying agar with the target strain. The rectangular SBS configuration facilitates factorial replication of dense microbial colony arrays for testing with multiple assays and assay conditions employing robotic colony pickers and pin tools. Opposed agar layers only slightly reduced the effectiveness for detecting growth inhibition from pure antibiotics compared to single-layer agar diffusion assays. The *Janus* plate enabled an automation-assisted workflow where a lone operator can effectively identify and accumulate bioactive soil bacterial strains within a few weeks. We also envisage the method’s utility for functional prescreening colonies of clones from genomic and metagenomic libraries or improved strains originating from mutagenized cells.

**Electronic supplementary material:**

The online version of this article (doi:10.1007/s10482-012-9746-7) contains supplementary material, which is available to authorized users.

## Introduction

Comparative genomics have demonstrated that the capacity of bacteria to produce secondary metabolites, antibiotics and other small bioactive molecules varies enormously, ranging from species having no capacity to those exceeding 30 different biosynthetic routes in cellularly differentiated bacteria with large genomes, e.g., myxobacteria and the actinomycetes (Donadio et al. 2007; Davies and Ryan 2011; Udwary et al. 2011). Antimicrobial activity can be a surrogate measurement for the ability of small molecules to interfere with or modulate cellular processes. Such molecules are defined as antibiotics when they negatively affect the growth of microbes (Davies 1990; Davies et al. 2006; Davies and Ryan 2011), or they can be more broadly defined as “perturbagens” if they modulate cell signaling pathways and cell growth and development in model organisms, including humans (Lamb 2007). Improved methods for rapid detection of functional molecules could expand the available chemistry pool and lead to discovery of new applications for small molecules in microbial and human cell biology.

The distinctive morphology of myxobacteria and actinomycetes can steer their preliminary selection for testing their potentially useful chemistry; however, the predominately featureless phenotypes of unicellular bacteria from soil, water and other substrata offer relatively few visual clues to guide the selection of biosynthetically capable strains (Gross and Loper 2009; Gram et al. 2010). However, direct prescreening of bacterial cells for their ability to produce antibiotics and perturbagens (Yim et al. 2007; Davies and Ryan 2011; Phelan et al. 2012) could circumvent laborious testing of shake-flask extracts from multitudes of bacterial strains and redirect resources to informed experimentation with the most biosynthetically capable species. That all biosynthetically capable bacteria would express their antibiotics in agar culture would be an unrealistic expectation. Nonetheless, direct microbial cell or colony assays can discriminate biosynthetically capable bacteria from those bacteria failing to produce antibiotics in vitro or those potentially lacking antibiotic biosynthetic pathways (Baltz et al. 2008; Gram et al. 2010; Wietz et al. 2010). Active strains can be funneled into enriched collections allowing resources for downstream fermentations and scale up to focus on prescreened bacteria with antibiotic phenotypes. The prescreening assay thus becomes a critical entry point into downstream processes and determines the frequency and characteristics of the antibiotic-producing bacteria selected for downstream study. Specificity and thresholds can be manipulated by either using overly sensitive or resistant assay or reporter strains, though caution must be exercised not to overload the process by causing artifacts or not to exclude interesting actives with highly insensitive strains.

Since the inception of the modern antibiotic era, many inventive agar diffusion methods have been developed to detect and measure the antimicrobial activity of microorganisms (Rios et al. 1988). These methods are unified around the observation that when a compound with biological activity diffuses through an agar layer homogeneously seeded with a target organism, and after incubation and growth of the target organisms, a clear to diffuse zone of inhibition (ZOI) radiates from the point of compound application. Agar diffusion assays remain popular because of their relative simplicity, low sample consumption and the capacity to test multiple compounds, concentrations, and mixtures from diverse sources in parallel against single or multiple microorganisms. Although not quantitative, these methods can be standardized by stringently controlling multiple factors (medium composition, microorganism sensitivity, extraction method, pH, solubility, etc.) to provide comparative data on antimicrobial spectra and potencies (Anonymous 1977; Bauer et al. 1988; Blom et al. 1997).

Agar overlay assays are well-established variants of agar diffusion assays that can rapidly prescreen environmental bacterial colonies (Nkanga and Hagedorn 1978; Furumai et al. 1993; Hayakawa et al. 2004; Shnit-Orland and Kushmaro 2009) or clones from metagenomic libraries (Brady 2007; Craig et al. 2010) for their ability to produce cell-penetrable antibiotics. In these methods, the target bacterium or yeast is homogeneously mixed in partially cooled agar and gently poured over the surface of a first layer previously inoculated with potential antimicrobial producer strains. The methods exploit tridimensional diffusion gradients; excreted antibiotics are detected by ZOIs forming on the upper side of the second agar layer (Gratia and Fredericq 1946; Gratia 1947; Nkanga and Hagedorn 1978; Williams et al. 1983; Hayakawa et al. 2004; Shnit-Orland and Kushmaro 2009). A frustrating shortcoming of these methods has been the disturbance and mixing of colonies upon adding the second layer, thus cross-contaminating and obscuring results, while complicating the isolation of the active bacterial colonies from the assay plate. To overcome cross contaminations, other variations of the agar overlay assay that incubate various kinds of inverted colony plates placed onto assay plates (Kekessy and Piguet 1970; Somkuti and Steinberg 2002) or that spot colonies or directly onto assay plates (Gram et al. 2010; Wietz et al. 2010) have been improvised.

To the best of our knowledge, the first device specifically designed to carry out an opposed two-layer agar assay, thus abbreviating the colony-to-active workflow, was the Lutri plate (former Lutri Plate, Inc. Starkville, MS, USA). It was a two-sided Petri plate-like device that sequentially created opposed agar layers for assaying the effects of microbes in one layer on the microbe growing in the other (Brown 1982; Colwell and Speidel 1985; Kang and Siragusa 1999). With this system, compounds excreted by organisms growing on one kind of agar diffused through the agar to affect an organism growing on another kind of agar in the opposing layer. An advantage of this method was that addition of a second assay layer did not disturb the first layer, while molecules could diffuse freely between both. However, to our knowledge, Lutri plates or any equivalent design are no longer manufactured or sold. Furthermore, the Lutri plate’s round design predated modern liquid handling and SBS plate robotics and would be incompatible with today’s high throughput screening equipment that are exclusively based on the SBS footprint.

A prototype double-sided culture plate, named a *Janus* plate after the two-faced Roman god, and inspired by the design of the Lutri plate was developed collaboratively with NUNC (www.nuncbrand.com). The plate enables growth of microorganisms on two opposed solid medium surfaces. The plate’s SBS footprint is the same as a rectangular NUNC OmniTray plate (96-well footprint, 128 × 85 mm). Like the Lutri plate, microbial antibiotics excreted into the first medium are detected by applying a second agar layer incorporating a target bacterium or yeast and observing ZOIs after incubation. Back-to-back agar layers configured in an OmniTray format offers several advantages over overlay techniques, including high test colony density, minimal cell disturbance by heating or pouring agar overlays, and compatibility with automated pipetting stations and colony picking robots.

Because most bacterially produced antibiotics act extracellularly, their activity can be detected at the bacterial colony interface or after its diffusion from the vicinity of a colony. Therefore, we sought a high throughput solution that would facilitate prescreening large populations of bacterial colonies in situations where the probability of antibiosis was infrequent. We compare the sensitivity of the *Janus* plate system with that of a single agar-layer antibiotic screening methods and describe a workflow for searching for antibiotics and perturbagens from bacterial colonies from environmental samples and that would also be appropriate for functional screening of clones from metagenomic libraries or mutagenized cells. To our knowledge, we describe the first high throughput prescreening tool employing the Lutri plate concept (Brown 1982) and hope that it will stimulate further efforts toward miniaturized high throughput screening from natural bacterial populations and from recombinant strains.

## Materials and methods

### Description and preparation of* Janus* and OmniTray plates

**Fig. 1:**
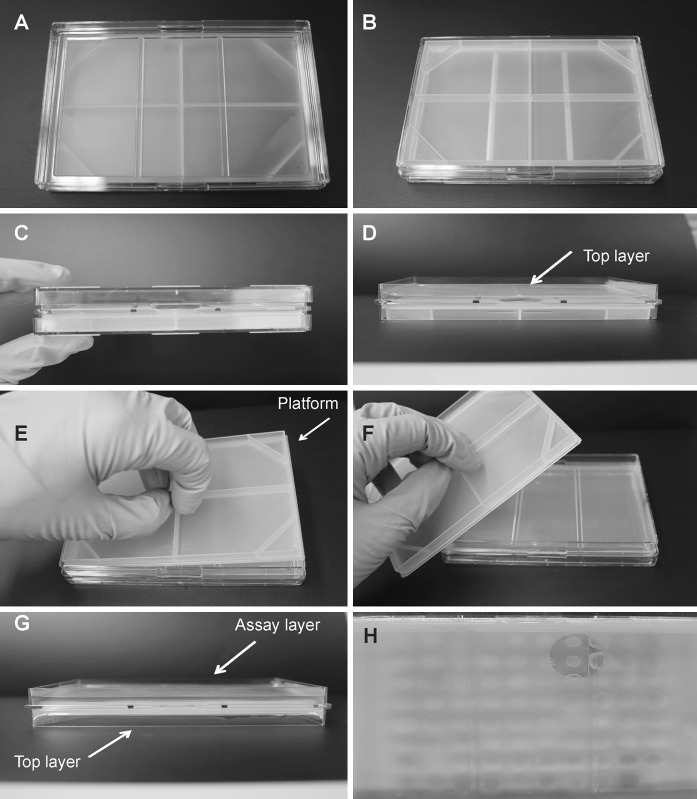
Description of *Janus* plates. *Top view* (**a**), *bottom view* (**b**) and *side view* with closed lids (**c**), view of first agar layer added to *Janus* plate. Isolation agar layer is poured onto top surface (**d**) supported by a removable plastic platform and producer organisms are incubated. After incubation, the plate is chilled to harden the agar, then inverted and the platform is removed (**e**, **f**). A second layer of media containing the target strain is poured across the lower surface of the first layer with lids removed (**g**). Zones of inhibition are visualized in the assay layer (**h**)

The *Janus* plate design (limited prototype manufactured by NUNC, Nalge Nunc International, Rochester, NY, USA) was based on the Lutri plate system (Brown 1982; Colwell and Speidel 1985) but was reengineered in polystyrene plastic to SBS standard dimensions to enable automated management (Fig. 1). A first medium layer (40 ml, 2 % agar) was poured onto one surface supported by a removable plastic platform (Fig. 1e, f). The agar was solidified around a rigid scaffold formed by two transverse ribs and a peripheral ledge (all 3 mm wide, Fig. 1). The first set of organisms was inoculated on this layer and incubated. After incubation, the plate was briefly chilled to 4 °C to harden the agar. The plate was inverted and the platform was removed (Fig. 1e, f). A second layer of media (30 ml, 1.5 % agar) containing the target strain was poured across the lower surface of the first layer.

The same media were added in parallel to OmniTray plates (NUNC, 40 ml, 2 % agar) for comparisons.

### Growth of antibiotic-producing and target strains and their media

The capacity of known antibiotic-producing *Streptomyces* strains for producing ZOIs was evaluated in *Janus* plates. Antibiotic-producing and target strains are described in Table 1. Reference strains from the Japan Collection of Microorganisms (www.jcm.riken.jp) reported to produce amphotericin B, tetracycline, oxytetracycline, or kanamycin were selected.

**Table 1:** Target and antibiotic-producing strains used in *Janus* plate demonstration experiments

Species	Strain number^a^	Assay function
*Candida albicans*	MY1055	Target, human pathogen
*Bacillus subtilis*	ATCC 6633	Target, human pathogen
*Pseudomonas aeruginosa*	PAO1	Target, human pathogen
*Staphylococcus aureus*	MB5393	Target, human pathogen
*Acinetobacter baumannii*	MB5973	Target, human pathogen
*Streptomyces nodosus*	JCM 4297 (type strain)	Produces amphotericin B
*Streptomyces rimosus*	JCM 4073 (type strain)	Produces oxytetracycline
*Streptomyces rimosus*	JCM 4667 (type strain)	Produces oxytetracycline
*Streptomyces varsoviensis*	JCM 4523 (type strain)	Produces oxytetracycline
*Streptomyces kanamyceticus*	JCM 4775 (type strain)	Produces kanamycin
*Streptomyces venezuelae*	JCM 4526 (type strain)	Produces chloramphenicol
*Burkholderia multivorans*	F-269,115	Produces unknown antifungal and antibacterial activity on many culture media

^a^Strains designated with MY, MB and F- are maintained in the Fundación MEDINA culture collection

The antibiotic-producing *Streptomyces* (Table 1) were revived from 10 % frozen glycerol stocks and grown in deepwell 96-well plates (Enzyscreen BV, www.enzyscreen.com) containing GYM broth (glucose 4 g, yeast extract 4 g, malt extract 10 g, CaCO_3_ 2 g, pH adjusted to 7.2, per litre of H_2_O) for 4 days at 28 °C and 220 rpm on an orbital shaker. Liquid cultures were replicated with a 96-pin plate replicator (Enzyscreen BV) onto the surface of 40 ml of four different 2 % agar-containing media (top layer of *Janus* plates): GYM with 2 % agar, DEF-15 (sucrose 4 g, NH_4_Cl 2 g, Na_2_SO_4_ 2 g, K_2_HPO_4_ 1 g, MgCl_2_·6H_2_O 1 g, NaCl 1 g, CaCO_3_ 2 g, 1 ml trace element solution [100 mg MnCl_2_·4H_2_O, 100 mg ZnCl_2_, 100 mg FeCl_2_ 4H_2_O, 50 mg NaI per l of H_2_O], pH adjusted to 7.0 per litre of H_2_O); FPY-12 (fructose 20 g, glucose 10 g, maltose 10 g, Bacto Peptone (Becton–Dickinson) 5 g, amicase 5 g, 1 ml trace elements [500 mg FeSO_4_·7H_2_O, 500 mg ZnSO_4_·7H_2_O, 100 mg MnSO_4_·H_2_O, 50 mg CuSO_4_·5H_2_O, 50 mg CoCl_2_·6H_2_O per litre of H_2_O], pH adjusted to 7.0); and KHC (dextrin from corn type I 20 g, ß-cyclodextrin 10 g, double-concentrated tomato paste 20 g, yeast extract 10 g, CoCl_2_·6H_2_O 5 mg, pH adjusted to 7.2 per litre of H_2_O).

To test for antifungal activity, a few colonies of *Candida albicans* were inoculated in 10 ml of Sabouraud dextrose broth (SDB, Becton–Dickinson) and incubated 18–20 h at 37 °C, 220 rpm on an orbital shaker. Optical density (OD_660_) of the overnight inoculum was measured and adjusted to 0.4, and 30 ml of the inoculum were added to 1 l of YNBD agar (6.7 g yeast nitrogen base, 1 g glucose, 15 g agar, distilled H_2_O 1 l) at 40 °C (Suay et al. 2000; Bills et al. 2008), which was poured onto the assay layer of the *Janus* plates.

To test for antibacterial activity, both *B. subtilis* and *P. aeruginosa* inocula were grown overnight in Luria broth (LB) at 37 °C, 220 rpm. The OD_600_ was adjusted to 0.4 in 30 ml of 1.5 % LB agar at 40 °C (Suay et al. 2000) and subsequently poured to the assay layer of *Janus* plates.

### Pure antibiotic assays

**Fig. 2:**
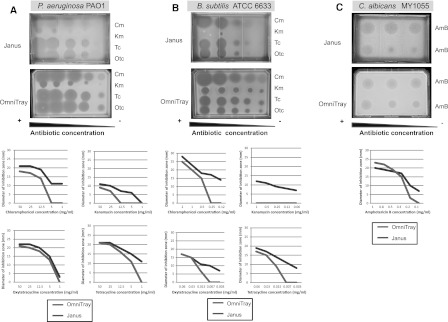
Different antibiotics dilution series applied to *P. aeruginosa* (**a**), *B. subtilis* (**b**) and *C. albicans* (**c**) growing in *Janus* and OmniTray plates. Photographs of assay plates (*above*) correspond to dose–response curves for each antibiotic (*below*). *Cm* chloramphenicol, *Km* kanamycin, *Tc* tetracycline, *Otc* oxytetracycline, and *AmB* amphotericin B

Two-fold serial dilutions of five antibiotics, four antibacterial (chloramphenicol, Cm; tetracycline, Tc; oxytetracycline, Otc; kanamycin, Km) and one antifungal (amphotericin B, AmB), were used to compare how pure antibiotics diffused in the two plate systems (Fig. 2). Cm was dissolved in ethanol, Tc, Otc, and AmB in 100 % DMSO and Km in H_2_O before preparing dilution series in ethanol, 20 % DMSO, and H_2_O respectively. Pathogens were unaffected by 20 % DMSO in parallel assays.

Forty ml of GYM with 2 % agar were poured on the top layer of the *Janus* plates. After solidifying, 5 μl of each dilution of antibiotic were spotted onto the media. The antibiotics were absorbed into the agar for approximately 1 h. The plates were inverted, the platform was removed and the assay layer was poured (30 ml of YNBD with 1.5 % agar containing *C. albicans* or 30 ml of LB with 1.5 % agar containing *B. subtilis* or *P. aeruginosa*).

In parallel, 40 ml of GYM with 2 % agar containing *C. albicans* or LB with 2 % agar containing *B. subtilis* or *P. aeruginosa* were poured into OmniTray plates. After solidification, 5 μl of the antibiotic dilutions were spotted on the surface in the same pattern as the *Janus* plates.

After overnight incubation at 37 °C, the plates were examined and the diameters of ZOIs were measured. All assays and measurements were made in triplicate.

### Producing strains assays

Forty ml of GYM, KHC, DEF-15 and FPY-12 with 2 % agar were poured on the top side of *Janus* plates. Once this layer solidified, producer strains grown in deepwell plates (see above) were inoculated onto the surface of the top agar layer with a 96-pin plate replicator. Plates were enclosed in plastic bags, and the strains were grown at 30 °C during five and ten days. After growth, the plates were inverted, the plastic platform was removed, and the assay layer was poured with either *C. albicans*, *B. subtilis*, or *P. aeruginosa* as described above. After overnight incubation at 37 °C, the plates were photographed and the diameters of ZOIs were measured. All assays and measurements were made in triplicate.

### Mass spectrometry and identification of antibiotics

To prepare agar culture samples for liquid chromatography mass spectrometry (LC–MS) analysis, 5 mm agar discs with antibiotic-producing bacterial colonies were cut from the upper agar layer with a sterile Transfer Tube (Spectrum Laboratories, Rancho Dominguez, California, USA). For each bacterium, three agar plugs were extracted with an equal volume of 20 % DMSO, and the extract was filtered to prior to LC–MS analysis.

Metabolite identification was undertaken in a two-step process. The first step used database matching with chromatographic retention time (RT), the UV–visible spectrum, and the positive and negative mass spectra from the extract components. Extract samples (1 μl) were analyzed with an Agilent (Santa Clara, CA) 1100 single quadrupole LC–MS, using a Zorbax SB-C8 column (2.1 × 30 mm), maintained at 40 °C and with a flow rate of 300 μl/min. Solvent A consisted of 10 % acetronitrile and 90 % water with 1.3 mM trifluoroacetic acid and ammonium formate, and solvent B was 90 % acetronitrile and 10 % water with 1.3 mM trifluoroacetic acid and ammonium formate. The gradient started at 10 % B and went to 100 % B in 6 min, kept at 100 % B for 2 min and returned to 10 % B for 2 min to initialize the system. Full diode array UV scans from 100 to 900 nm were collected in 4 nm steps at 0.25 s/scan. The eluting solvent was ionized using the standard Agilent 1100 electrospray ionization source adjusted to a drying gas flow of 11 l/min at 325 °C and a nebulizer pressure of 40 psig. The capillary voltage was set to 3,500 V. Mass spectra were collected as full scans from 150 to 1,500 *m/z*, with one scan every 0.77 s, in both positive and negative modes. The DAD spectra, retention time, positive and negative mass spectra of the active samples were searched with an in-house-developed application and compared to the UV–LC–MS data of known metabolites stored in a proprietary database where metabolite standard data had been obtained using the exact same LC–MS conditions as the samples under analysis (Vicente et al. 2009). Further database matches were confirmed by manually comparing UV–visible spectra.

In the case that no reasonable match was obtained, extracts were injected into another Agilent 1200 LC using the same LC parameters as above, and mass spectra were acquired on a Bruker maXis HR-TOF mass spectrometer (Bruker Daltonics GmbH, Bremen, Germany). Ionization of the eluting solvent was obtained using the standard maxis ESI source adjusted to a drying gas flow of 11 l/min at 200 °C and a nebulizer pressure of 40 psig. The capillary voltage was set to 4,000 V. Mass spectra were collected from 150 to 2,000 *m/z* in positive mode. Database matching compared the retention times and exact masses of sample extract components with the retention times and exact masses captured from authentic samples of known metabolites stored in a database and acquired under identical LC–MS conditions (Vicente et al. 2009) and manually with published data (Buckingham 2011).

### High throughput assay of soil bacteria

Soil samples were collected at different altitudes (1,125–2,311 m) in the Sierra Nevada National Park, Granada, Spain. Fresh soil samples (1 g) were dispersed in 100 ml of sterile diluent (VL70 medium without added growth substrates or vitamins) in 250-ml Erlenmeyer flasks by stirring with a magnetic bar for 30 min. These 10^−2^ diluted aliquots were serially diluted to 10^−6^. Then 100 μl of dilutions 10^−3^–10^−6^ were spread (five replicates at each dilution) onto the surface of the isolation media with sterile glass spreading rods. The media used for the isolations were gellan gum-solidified VL70 medium containing as growth substrates (1) d-xylose, 0.05 % (w/v); or (2) a mixture of peptone-casein, 0.025 % (w/v) each (Sait et al. 2002; Joseph et al. 2003; Davis et al. 2005). Both media were supplemented with 0.1 % w/v cycloheximide to inhibit fungi. Isolation plates were incubated at 18 °C and 60 % relative humidity in the dark. Bacterial colonies were picked from each dilution plate once a wk, during 5 weeks with an automated colony picking robot (Qpix2, Molecular Devices, New Milton, UK) and then transferred into 96-well tissue culture plates (Corning Inc., Corning, NY, USA) filled with 270 μl per well of R2A medium (Becton–Dickinson) (Fig. S1). After 2 weeks of incubation at 18 °C, 95-strain sets of soil strains were transferred from multiwell plates with a 96-pin plate replicator onto the top layer of the *Janus* plates containing 40 ml of R2A medium (Fig. S1). *Burkholderia multivorans* F-269,115, a highly antagonistic strain observed to consistently produce strong ZOIs against fungi and bacteria was inoculated in position A1 of assay plates as a positive control (Fig. S1). *Janus* plates were incubated at 18 °C for 5 days in plastic bags, and then the assay layer was poured with either *C. albicans*, *B. subtilis*, or *P. aeruginosa* as described above. After overnight incubation at 30 °C, the plates were examined for ZOIs and photographed. Active colonies were rescued from their corresponding master plates, and the assay was repeated in 24-colony arrays to confirm activity (Fig. S1). Before, proceeding with scale up, newly selected antibiotic-producing bacteria were frozen in glycerol 20 % (v/v) at −80 °C for long-term storage (Fig. S1).

### DNA extraction, 16S rRNA gene sequence analysis and identification of soil bacteria

Bacterial genomic DNAs were extracted by microwave lysis (Menon and Nagendra 2001). A few colonies of each strain were suspended in 750 μl of MilliQ water in a microcentrifuge tube and irradiated at the maximum power in a microwave oven with three pulses of 45 s alternating with a 30 s intervals. For amplification of partial 16S rRNA gene sequences, the primer pairs FD1 (5′-AGAGTTTGATCCTGGCTCAG-3′) and 1100R (5′-GGGTTGCGCTCGTTG-3′) were used. PCR mixtures were composed of 5.0 μl PCR buffer (10×), 4.0 μl MgCl_2_ (25 mM), 1.0 μl dNTPs (10 mM each), 1.0 μl each forward and reverse primers (10 μM), 0.3 μl Taq polymerase (5 U/μl Qbiogene) and 5.0 μl DNA solution in a total volume of 50 μl. The thermal cycling program was described previously (Pascual et al. 2010). PCR products were purified and sequenced using the above primer pairs at Secugen S.L. (Madrid, Spain). The nearest phylogenetic neighbors of the isolates were determined using the EzTaxon-e Database (http://eztaxon-e.ezbiocloud.net/) (Kim et al. 2012).

### Culture medium, fermentation and extraction of katanosins from *Lysobacter* sp.

Inoculum of a ZOI-producing strain *Lysobacter* sp. F-278,480 was prepared by transferring a loopful of surface growth from R2A agar into a tube with 5 ml R2A broth. The inoculated medium was incubated on a rotary shaker (220 rpm) at 28 °C for 48 h. The inoculum at 10 % v/v was transferred to 10 ml of fermentation medium (yeast extract 4 g, malt extract 10 g and dextrose 4 g, per l of H_2_O, pH adjusted to 7.0). The culture was fermented at 28 °C for 3 days on a rotary shaker at 220 rpm (Fig. S1). The fermentation was extracted with an equal volume of acetone amended with 20 % DMSO. Subsequently, the acetone was evaporated under a stream of N_2_, and the aqueous extract was assayed for growth inhibition of *B. subtilis*, *S. aureus*, *A. baumannii, P. aeruginosa* and *C. albicans* (Table 1). The active extract was analyzed by LC-HRMS as described above to identify the major metabolites that might contribute to the observed antibiosis.

## Results

### Comparative antibiotic assays

ZOIs from pure antibiotics against *P. aeruginosa* in *Janus* plates were comparable but proportionally smaller in *Janus* plates compared to those in OmniTray plates (Fig. 2a). The lowest concentration of Km (1 mg/ml) was not detectable in *Janus* plates.

Cm, Km, Tc and Otc were assayed at lower concentrations against the more sensitive *B. subtilis* (Fig. 2b) than against *P. aeruginosa* because the ZOIs overextended the plates and were unmeasurable. The ZOIs for Cm Tc, and Otc were slightly smaller in *Janus* plates than those observed in the OmniTrays at the equivalent concentrations. Detectable concentrations of Km apparently failed to penetrate into the assay layer of *Janus* plates (Fig. 2b).

Application of AmB at different concentrations in the first layer of *Janus* and in OmniTray plates caused comparable ZOIs against *C. albicans* (Fig. 2c). In *Janus* plates, the diameters of ZOIs were smaller than those detected in OmniTray plates at the lower concentrations (100 and 200 μg/ml), but at greater concentrations (between 300 and 1,000 μg/ml) the diameters were larger.

### Producer strains assay

Six *Streptomyces* reference strains known to produce the same antibiotics as used in the pure compound experiments (Table 1) were replicated in two rows onto the top layer of four different *Janus* plates containing four kinds of media (Fig. S2, legend). The cultures were grown during 5 and 10 days, and an assay layer incorporating the target strains was added (LB in the case of *B. subtilis* and *P. aeruginosa* and YNBD in the case of *C. albicans*, Fig. S2). Rather than each organism producing ZOIs corresponding to its reported antibiotic capacity, we observed an assortment of effects ranging from no growth, non-production, to potent and unexpected off-target antibiosis. As expected, the growth media and duration of incubation colony growth influenced the growth of strains and the intensity of antimicrobial activity caused by each antibiotic-producing strain. No single medium was optimal for detection of antibiosis from all strains.

*Streptomyces venezuelae* JCM 4526, the Cm producer, grew in the GYM liquid inoculum medium, but failed to grow in any of the solid agar media tested in the *Janus* plates and therefore its position on the growth layer was consistently vacant (Fig. S2). Both strains of *Streptomyces rimosus* and *S. varsoviensis*, although not previously reported to produce antifungal metabolites, were strongly active towards *C. albicans* on KHC, FPY-12, and GYM. Subsequent LC-HRMS analysis of agar colony extracts indicated that *S. rimosus* JCM 4073, not only produced oxytetracycline (C_22_H_24_N_2_O_9_, standard RT 0.73 min, *m/z* 460.1474, experimental RT 0.78, *m/z* 460.1473), but also produced a glycosylated polyene macrolide tentatively identified as rimocidin (C_39_H_61_NO_14_, calculated *m/z* 767.4092, experimental *m/z* 767.4097, RT 3.60 min with polyene-like UV spectrum) and a rimocidin analog, known as antibiotic CE108 (Pérez-Zuñiga et al. 2004) (C_39_H_61_NO_14_, calculated *m/z* 739.3779, experimental *m/z* 739.3788, RT 3.15 min with polyene-like UV spectrum) that could account for the potent antifungal activity. HRMS analysis of colony extracts of *S. kanamyceticus* JCM 4775 failed to detect kanamycin (C_18_H_36_N_4_O_11_, standard *m/z* 484.2380, RT 0.50 min), which along with its poor diffusion characteristics in the *Janus* plates (Fig. 2) would explain why no antibacterial ZO1 was observed from its colonies. *Streptomyces nodosus* JCM 4297, the AmB producer, generally grew poorly, although it inhibited *C. albicans* faintly on DEF-15 medium. Whether JCM 4297 contributed to the large ZOIs on GYM was questionable due the strong activity from the adjacent colonies. Most strains grown in DEF-15 medium produced poor antimicrobial activity against *P. aeruginosa* and *C. albicans*. Colonies of *S. rimosus* JCM 4667 and JCM 4067 (Otc producers) and *S. varsoviensis* (Otc producer) caused strong ZOIs against all three target strains (*C. albicans, P. aeruginosa* and *B. subtilis*) in KHC, DEF-15, and GYM, but *S. rimosus* JCM 4073 did not inhibit *P. aeruginosa* in KHC medium.

Incubation times of antibiotic-producing organisms influenced the sizes of ZOIs. However, depending on the specific medium-strain combination with larger or smaller ZOIs were evident at 5 or 10 days.

### Assays with soil bacteria arrays

**Fig. 3:**
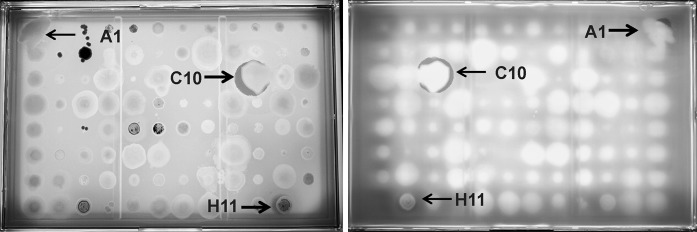
Zone of inhibition assay of a 96-colony array of wild-type bacteria grown on R2A agar against *Bacillus subtilis* grown in LB agar. Upper surface view of the growth layer (*left*) and bottom surface view of assay layer (*right*). A1, *Burkholderia multivorans* F-269,115 (positive control) with faint post incubation zone of inhibition; C10, *Lysobacter* sp. F-278,480, and H11, *Streptomyces* sp. F-278,462 with distinct zones of inhibition

A workflow was established starting with isolation of soil bacteria in Petri plates followed by robotic colony picking of bacterial colonies into 96-well master plates (Fig. S1). Master plates were replicated as 95-colony arrays directly into *Janus* plate assays with position A1 reserved for an antibiotic-producing positive control colony (Fig. 3).

VL70 isolation media supplemented with d-xylose or a mixture of peptone-casein as growth substrates enabled the isolation of bacterial strains belonging to a wide range of taxonomic groups, including actinobacteria, firmicutes, alpha-proteobacteria, beta-proteobacteria, gamma-proteobacteria and bacteroidetes (Table S1). The kinds of bacterial recovered were consistent with previous reports (Janssen et al. 2002; Sait et al. 2002; Joseph et al. 2003; Davis et al. 2005). Unknown and previously uncultured bacteria were also recovered at low frequencies. Two examples were strains F-278,959 and F-278,402, which showed only 97.2 and 96.2 % of 16S rRNA gene similarities with their closest cultivable neighbors, *Dyadobacter soli* (GQ241324) and *Rhizobium sphaerophysae* (FJ154088), respectively. The environmental strains were grown in R2A medium in the master plates and on the top layer of *Janus* plates because it was a relatively low nutrient medium, albeit more complex than VL70 medium. Parallel attempts to transfer colony arrays to high nutrient media, like trypticase soy agar, often resulted in poor replication, and in some cases, excessive growth and cell swarming. Nearly all bacteria recovered from the VL70 isolation medium were able to grow on R2A medium. Therefore R2A was used for transfer of colonies to 96-well master plates in order to provide cells for replication (Fig. S1) and for the growth medium for colony arrays on the top layer of the *Janus* plates.

After 5 or 10 days of incubation of the environmental strains and the control strain on the top layer of *Janus* plates, the target microorganisms were added to the assay layer. Microbial antibiotics or toxins excreted into the medium were detected as clear to diffuse ZOIs radiating from the antibiotic-producing colonies. Because low molecular weight molecules can diffuse freely between both layers, ZOIs could be detected on either the upper side of the first and second agar layers (Fig. 3). However, some false positives caused by colony shadows were evident, but they could be discounted by illuminating true ZOIs on the outer surface of the assay layer with a fluorescent transilluminator.

During preliminary assays of 7,315 colonies, the average number of antibiotic-producing soil isolates causing ZOIs with at least one of the three-pathogen bioassay (the same colony array on each of three *Janus* plates with a different target pathogens) and totaling 21,945 assays, was 1.6 %. The numbers of antibiotic-producing filamentous actinobacteria and non-filamentous bacteria detected in *Janus* plates were 30 (27.7 %) and 88 (72.3 %) respectively. As expected, *B. subtilis* ATCC 6633 and *C. albicans* MY1055 were more sensitive targets than *P. aeruginosa* strain PAO1. No additional antibiotic-producing bacteria were observed or recovered when wild-type strain arrays were incubated for longer periods (10 days) on the top layer of *Janus* plates.

Figure 3 illustrates a wild-type bacteria array assayed in a *Janus* plate. A set of environmental strains were arrayed in a 95-matrix, and position A1 was inoculated with a positive control *Burkholderia multivorans* F-269,115. The target strain in this assay was *B. subtilis*. After overnight assay incubation, three ZOIs were scored, the positive control (A1), and two wild-type strains identified as *Lysobacter* sp. F-278,480 (C10) and *Streptomyces* sp. F-278,462 (H11). The ZOI associated with the *Lysobacter* sp. F-278,480 was larger than those caused by *B. multivorans* F-269,115 (positive control) and *Streptomyces* sp. F-278,462. Colonies of some swarming and gliding bacteria sometimes extended across the R2A agar layer, colonizing larger areas or neighboring bacterial colonies. For example, *Lysobacter* sp. F-278,480 glided toward a neighbor colony, surrounding and lysing it (Fig. 3), a characteristic trait of *Lysobacter* species. The *Lysobacter* and *Streptomyces* strains were rescued from the master plate, and re-inoculated under the same physical and nutritional conditions in a 24-colony array on a second *Janus* plate assay (see workflow Fig. S1). The confirmed active strain of *Lysobacter* sp. F-278,480 was fermented in 10-ml tube fermentations and extracted with acetone. The aqueous extract amended with 20 % DMSO was assayed for antimicrobial activity and analyzed by LC-HRMS. Organic extracts inhibited the growth of *B. subtilis*, *S. aureus* MRSA and *C. albicans*, but not *P. aeruginosa* PAO1 nor *A. baumannii*. Dereplication by LC-HRMS (1 ppm mass accuracy as defined by an internal standard) and comparison with published compounds led to the tentative identification of katanosins A (C_87_H_95_N_15_O_17_, calculated *m/z* 1261.7030, experimental *m/z* 1261.7028, RT 2.98 min) and B (C_58_H_97_N_15_O_17_, calculated *m/z* 1275.7187, experimental *m/z* 1275.7188, RT 3.08 min). Therefore, antimicrobial activity against Gram positive bacteria was likely attributable to katanosins, also known as lysobactins, cyclic depsipeptides that target bacterial cell wall biosynthesis (Bonner et al. 1988; O’Sullivan et al. 1988; Guzman-Martinez et al. 2007). The extracts components responsible for the *C. albicans* growth inhibition are under investigation.

## Discussion

Because natural products discovery is a factorial process depending the interaction among microorganisms × growth conditions × assays (Bode et al. 2002), the ability to replicate colonies across conditions and assay them against different target cells significantly increases the opportunities to detect a metabolite-producing colony. Previously, we have developed prescreening solutions, referred to as nutritional arrays, aimed towards building collections of microorganisms enriched for their capacity to produce antibiotics by employing microfermentation systems for fungi and actinomycetes that enabled testing of strain arrays across many media conditions (Bills et al. 2008; Genilloud et al. 2011; Phillips et al. 2011; Roemer et al. 2011). *Janus* plates can be viewed as a progression towards simplification of high throughput screening systems for bioactive secondary metabolite producing strains that further miniaturizes and eliminates processing steps, while increasing the capacity to evaluate many organisms while varying nutritional, environmental, or assay permutations. This automation-assisted workflow can assess a wide range of bacterial taxonomic groups with the goal of increasing the chances of finding new extracellularly produced cell-perturbing molecules (Fig. S1). The proposed screening paradigm is conceptually similar to an industrialized high throughput system implanted at Cubist Pharmaceutical where large numbers actinomycetes isolates were encapsulated in alginate beads and nascent colonies were massively screened on solid agar surfaces against pathogen strains with engineered resistance genes (Baltz et al. 2008). However detection of antibiotic-producing *Streptomyces* species from soils would be trivial in *Janus* plates (Figs. 3, S2). The workflow’s utility can be better appreciated by the fact that a single operator (J.P.) could recover and detect a katanosin-producing strain of *Lysobacter*, a bacterial genus known to be rich in antibiotics but infrequently recovered from soils (Christensen 2005) in a few weeks.

We view the use of the *Janus* plates as a way to quickly focus valuable fermentation resources on strains with the highest potential for bioactive metabolites, rather than as replacement for traditional discovery-scale fermentations in tubes or flasks. A similar approach has been applied to survey marine bacteria for antibiosis. Thousands of single marine bacterial isolates and replicated sets of marine bacterial isolates were prescreened for inhibitory activity against a single target strain, *Vibrio anguillarum*, in a single assay condition (glucose and casamino acids agar at 25 °C) to select potential antibiotic-producing marine bacteria (Gram et al. 2010; Wietz et al. 2010, 2012). Only strains reproducing the activity on scale up in small volume fermentations were further studied by biological activity and high resolution MS profiling. Prescreening with the *Janus* plates offers a similar economy of throughput but can be widen the window of opportunity for detecting antibiotics or perturbagens by adding more target organisms and organism-specific growth conditions. Similar economy of scale was gained by using microfermentation systems, especially in the ability to replicate master sets of strains across nutrition or environmental arrays of conditions (Bills et al. 2008; Genilloud et al. 2011). However, the *Janus* plate system reduces the assay to a near proximity interaction between the excreting colony and the target strain, the need for fermentation and extract processing are postponed until the value of the active microorganisms are confirmed. The workflow outlined herein (Fig. S1) would be advantageous for screening microorganisms in situations where the antibiotic detection is infrequent, e.g., screening for functional molecules against especially when resistant target organisms are sought, e.g., drug resistant *Pseudomonas* species. Other applications for functional screening of clones from metagenomic libraries (Brady 2007; Craig et al. 2010) or mutation screening during process improvement can be envisaged as well.

Despite the obvious gains in efficiency, throughput and assay flexibility, the *Janus* plate system, like any in vitro growth system, has its shortcomings. Firstly, growth and an accumulation of a minimal biomass is a prerequisite for detecting a microbial colony’s biosynthetic products, and not all bacteria grow well on all agar surfaces, nor do all cells transfer well with pin tools (Fig. S2). Cell-bound antibiotics would not be detected. The dual agar layer increases the diffusion distance, thus as demonstrated in the pure antibiotics experiments, some sensitivity is lost. The system relies on excretion of sufficient antibiotic concentrations for visual detection of growth inhibition or modified phenotypes; therefore, target strain sensitivity and its optical properties are critical. To increase visual sensitivity, one could imagine using pigmented, fluorescent or luminescent strains or reporter strains that change phenotype when a specific target is affected. Antibiotics with poor diffusion characteristics or that spontaneously degrade may be missed, such as was demonstrated with application of pure kanamycin. The composition of the growth layer and incubation will likely profoundly affect antibiotic biosynthesis, and failure to detect antibiosis in one agar medium could be compensated by replicating colony arrays onto multiple media for growth for at different temperatures and times.

In preliminary experiments, we have seen only a few instances of suspected heat-labile antibiosis from colonies, presumably caused by lytic enzymes. Possibly sharp changes in pH and excretion of siderophores could also cause false positives that resemble antibiosis. So far, we have not seen evidence of antibiosis induced by the close proximity of two different bacterial species, but conceivably, in high density colonies, interspecific colony interactions could stimulate production of metabolites not produced in strictly axenic culture, thus introducing false positives. On the other hand, the *Janus* plates could facilitate a screening strategy directed at monitoring the antibiotics responses to multiple combinations of bacteria grown in close proximity. Some organisms, e.g., *Streptomyces* species, will likely produce multiple antibiotics. Therefore the use of a *Janus* plate assay to search for specific antibiotic classes or antibiotics with specific modes of action would need to be carefully designed and interpreted.

The *Janus* dual agar layer is somewhat frail and must be handled and incubated carefully. When colony arrays were grown during several days, plates were enclosed in plastic bags to prevent desiccation which leads to agar shrinkage and collapse of the growth layer. In our wild colony prescreening example, bacteria from high elevation soils were incubated at 18 °C, so desiccation was inconsequential, but prolonged incubation at warm temperatures (>25 °C) could be problematic. Warmer temperature also will promote swarming and gliding in some kinds of bacteria, and at high colony densities, cross contamination can occur. The double agar layer also can obscure detection and interpretation of weak ZOIs, and ZOIs are most conservatively observed by inspecting the upper surface of the assay layer. Also, large ZOIs may span several adjacent colonies; in this case, all colonies from within the ZOI should be rescued and reconfirmed in a second low density assay. Finally, we advise caution in using two different agar layers that may be nutritionally incompatible or toxic to the target organisms, e.g., high osmotic concentrations of salts or sugars that would inhibit growth of the target pathogen.

## Conclusions

Antimicrobial assays in two opposed agar layers formed in *Janus* plates behaved similarly to parallel antibiotic assays in singe agar layers in OmniTray plates. The *Janus* plates facilitated a workflow for prescreening large populations of wild colonies of bacteria for cell-perturbing small molecules, e.g., antibiotics. Bacterial colonies recovered on specialized isolation media can be transferred to 96-wells plates with a robotic colony picker. Once grown in 96-well master plates, sets of colonies can be factorially replicated onto *Janus* plates with different nutrient media in the top growth layer. A separate growth layer means plates can be incubated at varied time intervals and conditions to stimulate excretion of bioactive molecules. Once grown, replicated colony arrays can be assayed against multiple target cells in the opposed assay layer. ZOIs or other phenotypic changes in the target organisms guide selection of those bacteria capable of excreting cell-perturbing small metabolites which are then accumulated for more elaborate fermentations aimed at stimulating, evaluating and identifying those molecules.

## Electronic supplementary material

Below is the link to the electronic supplementary material.

Supplementary material 1 (PDF 3495 kb)

Supplementary material 2 (PDF 341 kb)

Supplementary material 3 (DOCX 15 kb)
