# Supplementary material for: Prescreening bacterial colonies for bioactive molecules with Janus plates, a SBS standard double-faced microbial culturing system
Source: Antonie Van Leeuwenhoek. 2012 May 5;102(2):361–74. doi: 10.1007/s10482-012-9746-7 (PMC3397223; doi:10.1007/s10482-012-9746-7)
Supplement: Supplementary file 1 — Supplementary material 1 (PDF 3495 kb) [file 10482_2012_9746_MOESM1_ESM.pdf]

Media

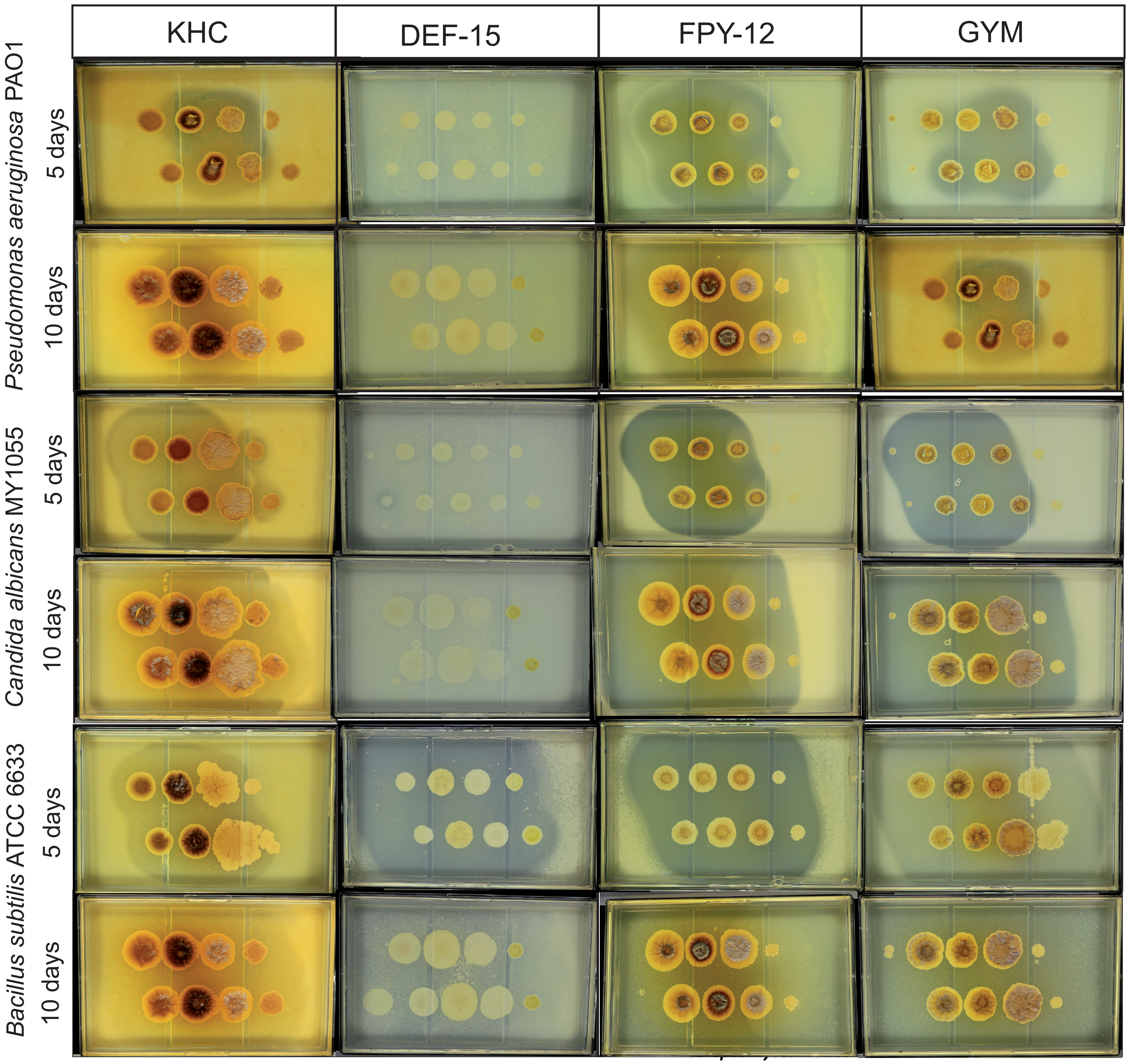

Innoculation pattern

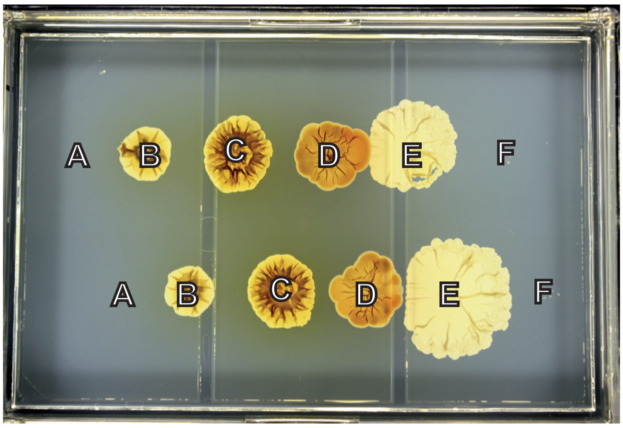

A. *Streptomyces nodosus* JCM 4297
B. *Streptomyces rimosus* JCM 4073
C. *Streptomyces rimosus* JCM 4667
D. *Streptomyces varsoviensis* JCM 4523
E. *Streptomyces kanamyceticus* JCM 4775
F. *Streptomyces venezuelae* JCM 4526

Fig. S2. Zones of inhibition caused by antibiotic producers against three different indicator strains in *Janus* plates. Producers were incubated in four media (KHC, DEF-15, FPY-12 and GYM) during five and ten days, as described in Material and methods. The inoculation pattern of the producing strains is indicated on a plate where colonies were replicated onto LB agar (bottom).
