# Supplementary material for: Prescreening bacterial colonies for bioactive molecules with Janus plates, a SBS standard double-faced microbial culturing system
Source: Antonie Van Leeuwenhoek. 2012 May 5;102(2):361–74. doi: 10.1007/s10482-012-9746-7 (PMC3397223; doi:10.1007/s10482-012-9746-7)

Figure S1. Workflow for prescreening soil bacteria for antibiotics and cell-perturbing molecules, confirming activity, scaling up active bacteria and preparing extract collection.

Step 1. Transfer individual colonies from isolation plates to master plate (96- well tissue culture plate) with RA2 agar using an automated colony picking robot

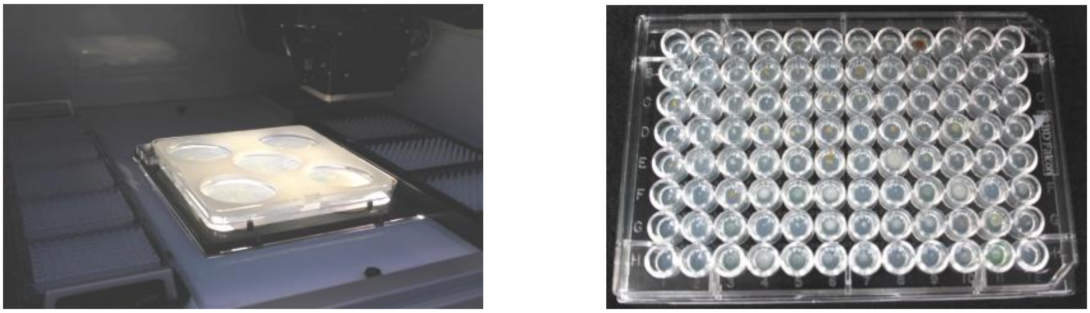

Step 2. Master plate replication onto the first layer of *Janus* plates (one for each bioassay) using a 96-pin plate replicator

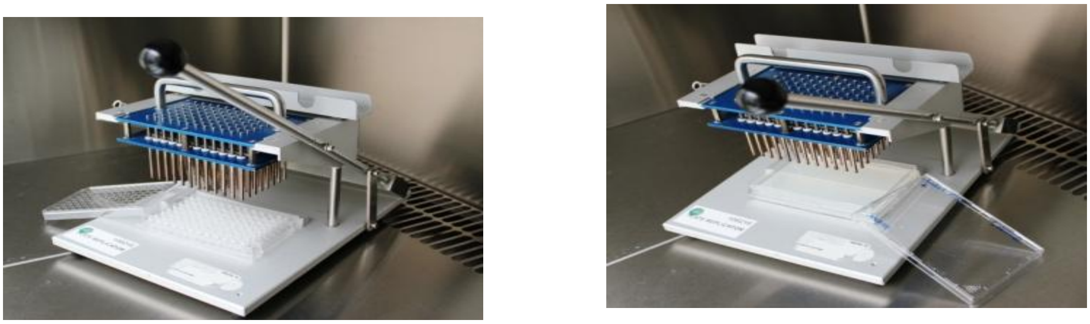

Step 3. Grow environmental colonies at 18 ºC, 5 d. Subsequently, invert *Janus* plate, remove support and inoculate the second layer with an indicator strain (e.g., *B. subtilis*, *P. aeruginosa* or *C. albicans*) . ZOIs are detected after 24 h at 33-37 ºC

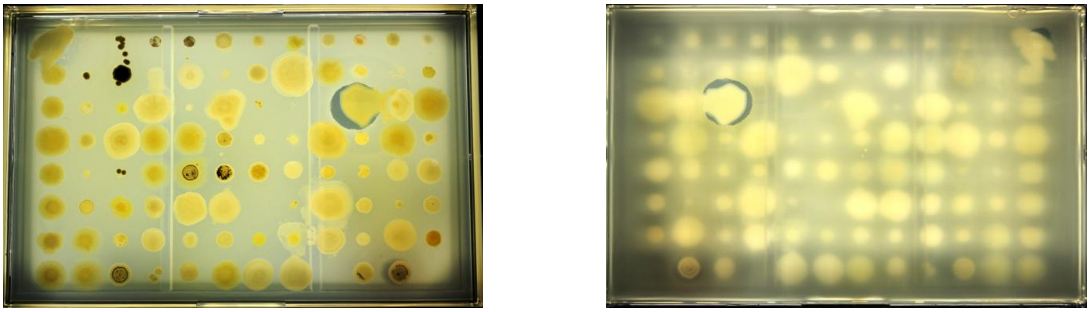

Figure S1 continued. Workflow for prescreening soil bacteria for antibiotics and cell-perturbing molecules, confirming activity, scaling up active bacteria and preparing extract collection.

Step 4. Manually rescue active colonies from their corresponding master plates, and repeat the assay in 24-colony arrays to confirm activity

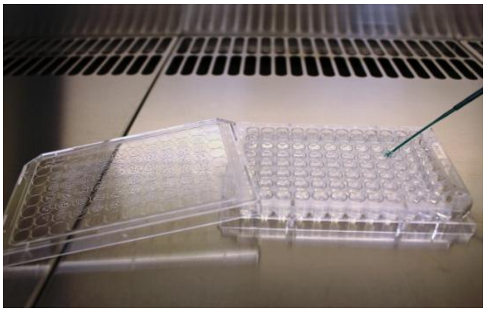

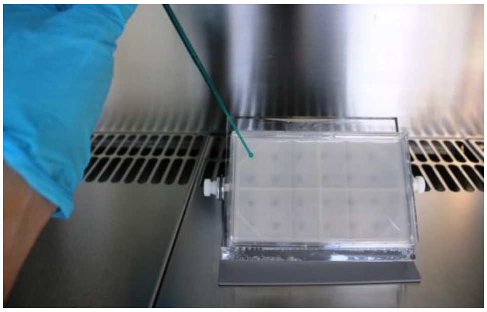

Step 5. Grow pre-selected colonies at 18 ºC, 5 d. Subsequently, invert *Janus* plate, remove support and inoculate the second layer with an indicador strain (*B. subtilis*, *P. aeruginosa* or *C. albicans*). ZOIs are detected after 24 h at 33 ºC

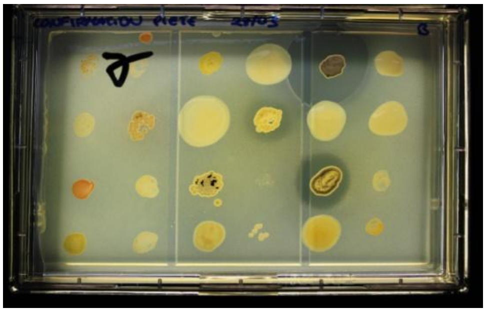

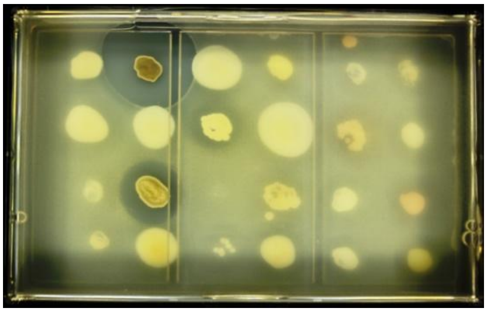

Step 6. Collection of confirmed bioactive bacteria identified by partial 16S rRNA sequences and preserved in 20% (v/v) glycerol at -80 ºC

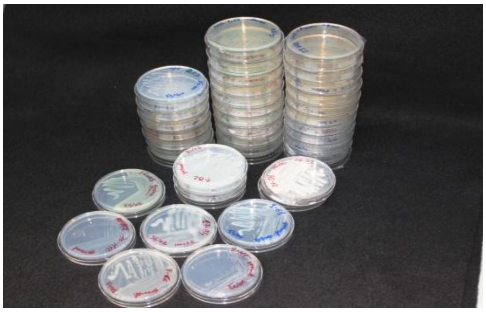

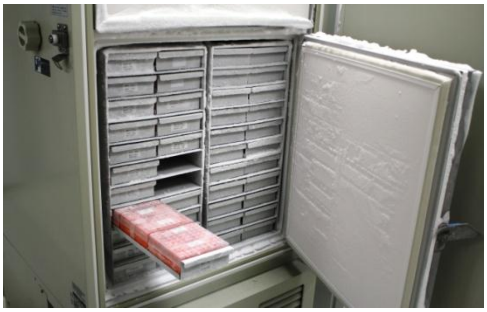

Figure S1 continued. Workflow for prescreening soil bacteria for antibiotics and cell-perturbing molecules, confirming activity, scaling up active bacteria and preparing extract collection.

Step 6. Fermentation in four different media (10 ml) and two different temperatures on a rotary shaker

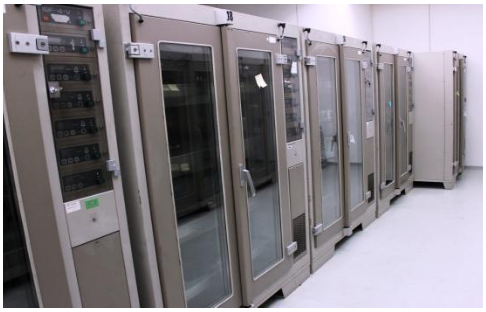

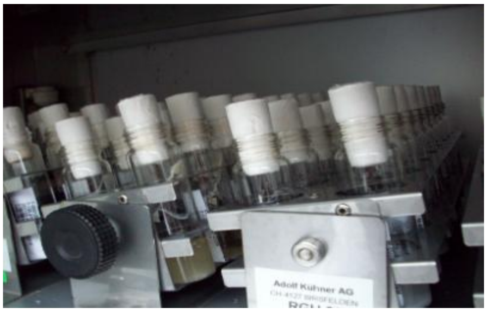

Step 7. Extract fermentations with equal volume of acetone, add 20% DMSO, evaporate acetone and dispense aqueous extracts in assay plates, creating a collection of enriched bioactive bacterial extracts. Store extracts at -20 C until ready for assay.

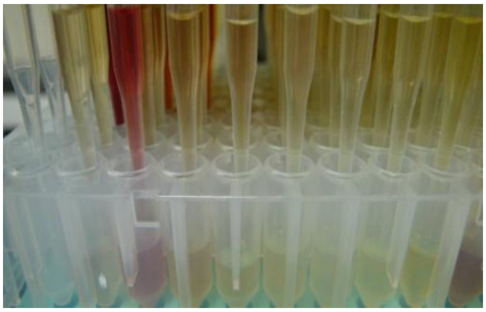

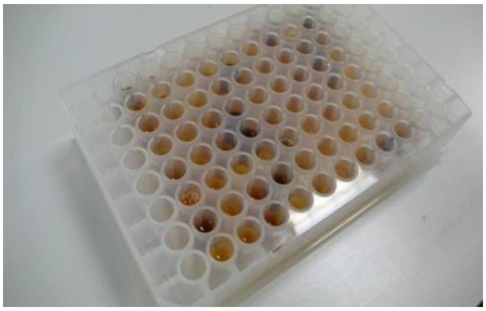

Supplement: Supplementary file 2 — Supplementary material 2 (PDF 341 kb) [file 10482_2012_9746_MOESM2_ESM.pdf]
