# Supplementary material for: Prescreening bacterial colonies for bioactive molecules with Janus plates, a SBS standard double-faced microbial culturing system
Source: Antonie Van Leeuwenhoek. 2012 May 5;102(2):361–74. doi: 10.1007/s10482-012-9746-7 (PMC3397223; doi:10.1007/s10482-012-9746-7)
Supplement: Supplementary file 3 — Supplementary material 3 (DOCX 15 kb) [file 10482_2012_9746_MOESM3_ESM.docx]

Table S1. Taxonomic position of 71 environmental strains isolated from the a single soil sample from the Sierra Nevada National Park, Spain. Strains are listed alphabetically according to their nearest neighbour based on partial 16S rRNA gene sequences.

| **Closest neighbour^a^** | **Similarity (%)** | **Taxonomic group** |
| --- | --- | --- |
| *Aeromicrobium ginsengisoli* | 99.9 | Actinobacteria |
| *Agreia pratensis* | 99.8 | Actinobacteria |
| *Arthrobacter globiformis* | 99.6 | Actinobacteria |
| *Arthrobacter oxydans* | 98.6 | Actinobacteria |
| *Arthrobacter pascens* | 99.2 | Actinobacteria |
| *Arthrobacter phenanthrenivorans* | 98.8 | Actinobacteria |
| *Blastococcus aggregatus* | 98.4 | Actinobacteria |
| *Cellulomonas aerilata* | 98.1 | Actinobacteria |
| *Cellulomonas cellasea* | 99.8 | Actinobacteria |
| *Cellulomonas terrae* | 98.4 | Actinobacteria |
| *Leifsonia kafniensis* | 97.9 | Actinobacteria |
| *Microbacterium flavescens* | 98.7 | Actinobacteria |
| *Microbacterium phyllosphaerae* | 98.8 | Actinobacteria |
| *Microbacterium pygmaeum* | 98.0 | Actinobacteria |
| *Microbacterium terricola* | 98.2 | Actinobacteria |
| *Modestobacter versicolor* | 99.3 | Actinobacteria |
| *Mycobacterium aurum* | 98.9 | Actinobacteria |
| *Mycobacterium canariasense* | 96.9 | Actinobacteria |
| *Mycobacterium hodleri* | 98.5 | Actinobacteria |
| *Nocardia fluminea* | 99.5 | Actinobacteria |
| *Nocardioides ganghwensis* | 96.9 | Actinobacteria |
| *Nocardioides hankookensis* | 97.3 | Actinobacteria |
| *Nocardioides hwasunensis* | 97.6 | Actinobacteria |
| *Nocardioides iriomotensis* | 96.9 | Actinobacteria |
| *Nocardioides pyridinolyticus* | 98.3 | Actinobacteria |
| *Streptomyces anulatus* | 99.1 | Actinobacteria |
| *Streptomyces asterosporu* | 98.8 | Actinobacteria |
| *Streptomyces canus* | 98.4 | Actinobacteria |
| *Streptomyces cinereorectus* | 100 | Actinobacteria |
| *Streptomyces ciscaucasicus* | 98.9 | Actinobacteria |
| *Streptomyces corchorusii* | 98.2 | Actinobacteria |
| *Streptomyces cyaneofuscatus* | 99.2 | Actinobacteria |
| *Streptomyces durhanensis* | 98.1 | Actinobacteria |
| *Streptomyces eurythermus* | 98.8 | Actinobacteria |
| *Streptomyces fildesensis* | 97.8 | Actinobacteria |
| *Streptomyces galilaeus* | 90.7 | Actinobacteria |
| *Streptomyces globisporus* | 99.4 | Actinobacteria |
| *Streptomyces griseoplanus* | 99.0 | Actinobacteria |
| *Streptomyces griseorubiginosus* | 99.1 | Actinobacteria |
| *Streptomyces microflavus* | 99.6 | Actinobacteria |
| *Streptomyces novaecaesareae* | 100 | Actinobacteria |
| *Streptomyces plumbiresistens* | 94.9 | Actinobacteria |
| *Streptomyces tauricus* | 98.7 | Actinobacteria |
| *Williamsia limnetica* | 99.4 | Actinobacteria |
| *Brevibacterium frigoritolerans* | 99.8 | Firmicutes |
| *Paenibacillus alginolyticus* | 97.9 | Firmicutes |
| *Paenibacillus lautus* | 99.4 | Firmicutes |
| *Psychrobacillus psychrodurans* | 99.5 | Firmicutes |
| *Ancylobacter dichloromethaniscus* | 97.3 | Alphaproteobacteria |
| *Bradyrhizobium cytisi* | 96.7 | Alphaproteobacteria |
| *Devosia neptuniae* | 99.6 | Alphaproteobacteria |
| *Methylobacterium brachiatum* | 99.9 | Alphaproteobacteria |
| *Methylobacterium marchantiae* | 99.8 | Alphaproteobacteria |
| *Methylobacterium soli* | 97.2 | Alphaproteobacteria |
| *Microvirga aerilata* | 98.2 | Alphaproteobacteria |
| *Phyllobacterium bourgognense* | 98.7 | Alphaproteobacteria |
| *Phyllobacterium ifriqiyense* | 99.9 | Alphaproteobacteria |
| *Phyllobacterium trifolii* | 97.7 | Alphaproteobacteria |
| *Rhizobium herbae* | 99.6 | Alphaproteobacteria |
| *Rhizobium leguminosarum* | 99.3 | Alphaproteobacteria |
| *Skermanella aerolata* | 99.3 | Alphaproteobacteria |
| *Sphingomonas faeni* | 99.9 | Alphaproteobacteria |
| *Burkholderia graminis* | 99.1 | Betaproteobacteria |
| *Massilia consociata* | 95.2 | Betaproteobacteria |
| *Moraxella osloensis* | 99.6 | Gammaproteobacteria |
| *Stenotrophomonas rhizophila* | 99.9 | Gammaproteobacteria |
| *Xanthomonas bromi* | 97.7 | Gammaproteobacteria |
| *Xanthomonas gardneri* | 98.8 | Gammaproteobacteria |
| *Hymenobacter perfusus* | 94.9 | Bacteroides |
| *Flavobacterium frigidimaris* | 99.7 | Bacteroidetes |

^a^Based on sequence similarity searches in the EzTaxon-e Database (http://eztaxon-e.ezbiocloud.net/).
